# Lactate versus Phosphate as Biomarkers to Aid Mechanical Circulatory Support Decisions in Patients with Out-of-Hospital Cardiac Arrest and Return of Spontaneous Circulation

**DOI:** 10.3390/diagnostics13091523

**Published:** 2023-04-24

**Authors:** Dragos Andrei Duse, Fabian Voß, Laura Heyng, Georg Wolff, Christine Quast, Daniel Scheiber, Patrick Horn, Malte Kelm, Ralf Westenfeld, Christian Jung, Ralf Erkens

**Affiliations:** 1 Department of Cardiology, Pulmonology, and Vascular Medicine, University Hospital Düsseldorf, Medical Faculty, Heinrich Heine University Düsseldorf, 40225 Düsseldorf, Germany; 2 Cardiovascular Research Institute Düsseldorf (CARID), University Hospital Düsseldorf, Medical Faculty, Heinrich-Heine-University Düsseldorf, 40225 Düsseldorf, Germany; 3 Abiomed Europe GmbH Europe, Neunhofer Weg 3, 52074 Aachen, Germany

**Keywords:** phosphate, mechanical-circulatory-support, out-of-hospital cardiac arrest, survival, ROSC

## Abstract

Aims: Identifying patients who may benefit from mechanical circulatory support (MCS) after out-of-hospital cardiac arrest (OHCA) and return of spontaneous circulation (ROSC) remains challenging; thus, a search for helpful biomarkers is warranted. We aimed to evaluate phosphate and lactate levels on admission regarding their associations with survival with and without MCS. Methods: In 224 OHCA patients who achieved ROSC, the initial phosphate and lactate levels were investigated to discriminate in-hospital mortality by receiver operating characteristic (ROC) curves. According to the Youden Index (YI) from the respective ROC, the groups were risk stratified by both biomarkers, and 30-day mortality was analyzed in patients with and without MCS. Results: Within the entire collective, MCS was not associated with a better chance of survival. Both phosphate and lactate level elevations showed good yet comparable discriminations to predict mortality (areas under the curve: 0.80 vs. 0.79, *p* = 0.74). In patients with initial phosphate values > 2.2 mmol/L (>YI), 30-day mortality within the MCS cohort was lower (HR 2.3, 95% CI: 1.4–3.7; *p* = 0.0037). In patients with lower phosphate levels and groups stratified by lactate, 30-day mortality was similar in patients with and without MCS. Conclusions: We found a significant association between survival and MCS therapy in patients with phosphate levels above 2.2 mmol/L (Youden Index), and a similar discrimination of patient overall survival by lactate and phosphate. Prospective studies should assess the possible independent prognostic value of phosphate and its clearance for MCS efficiency.

## 1. Introduction

Out-of-hospital cardiac arrest (OHCA) remains a frequent cause of death in western countries, with an incidence of 1 per 1000 [1]. While the return of spontaneous circulation (ROSC) is mandatory for survival, several factors, such as bystander CPR, an initial shockable rhythm, or a high chest compression fraction, are key features previously associated with a higher chance of ROSC [2,3]. Following OHCA and after achieving ROSC, a severe complication shown by numerous patients is a deteriorating hemodynamic situation, ultimately worsening the chance of survival and neurological outcome [4,5]. To date, algorithms to predict the overall outcome are imprecise in light of a possible threat of hemodynamic instability following ROSC. Whereas several parameters, such as time to ROSC, initial rhythm, bystander CPR, or cause of CA, were associated with survival [6,7], forecasting an immediate cardio-circulatory deterioration remains challenging.

The Society for Cardiovascular Angiography and Interventions (SCAI) recently released an updated shock stage classification in an attempt to stratify patients correctly according to their clinical symptoms, biochemical markers, and hemodynamics [8]. The classification mentioned Mechanical Circulatory Support (MCS) as a bail-out strategy to restore stable circulation by assisting cardiac function. In OHCA patients under ongoing CPR, MCS seems highly valued as it might counteract metabolic derangements induced by long-lasting CPR and circulatory instability, which is also mirrored by the fact that it is recommended in the 2021 ERC Guidelines as a rescue therapy [9,10,11].

Whereas the SCAI scale facilitated a shock stage categorization, clear criteria for physicians to decide which patients should receive MCS are missing. Clinically used scores for risk stratification in patients with cardiogenic shock after OHCA show only moderate accuracy in patients treated with MCS [12]. The American Heart Association (AHA) currently recommends an individual decision for every shock patient after consulting with the shock team based on the best existing information at the time of the decision [13]. However, an evidence-based, generally available recommendation for physicians to use MCS is currently open for question [5]. Identifying prognostication parameters that are fast and feasible to determine might improve the treatment algorithms, as they would augment the best existing information at the time. Even in light of the benefits of circulatory support, MCS remains an invasive procedure, and several trials showed no benefit from MCS in cardiogenic shock (CS) patients [14,15,16], reflecting that MCS should not be routinely used in all patients with CS.

Lactate, a known marker for tissue hypoxia [17], has previously been associated with survival following OHCA [18]. Its inclusion in the shock group stratification by the SCAI highlights its importance in hemodynamic alterations [8], making it probably the most important prognostic biomarker in intensive care. Additionally, lactate levels seem to reflect 30-day mortality in patients with cardiogenic shock treated with MCS [19,20], suggesting their prognostic value for MCS implementation.

We previously found a moderate correlation between lactate and phosphate levels in CA patients [21]. In addition, we and others found initial serum phosphate level elevations associated with a higher risk of mortality after CA [21,22,23]. Serum phosphate plays a crucial role in cellular energy production [24], and it maintains mitochondrial membrane potential [25]. However, it contributes to the acidosis following CA [26,27], making it a surrogate biomarker for microperfusion and tissue vitality. In this retrospective study, we hypothesized that initial phosphate level elevations might discriminate similarly well as lactate those patients after ROSC, in whom MCS would be associated with a greater chance of survival.

## 2. Methods

### 2.1. Study Design and Population

This study was a retrospective observational cohort, single-center study performed at the University Hospital in Duesseldorf. The analysis screened 388 OHCA patients who were treated between 2016 and 2019. Inclusion criteria were non-traumatic OHCA with ROSC, aged ≥18 years, and with recorded serum phosphate at admission. Data were collected from the clinical data information systems (*Medico* (Cerner GmBh Berlin, Germany) and *PEGASOS* (Nexus/Marabu GmBh Berlin, Germany)).

This study was approved by the ethics committee of the Medical Faculty of the Heinrich-Heine-University in Duesseldorf, Germany, with the reference number 2018-153-KFogU. Due to the retrospective nature of our study, informed consent was waived by the ethics committee.

Based on the entire collective, we first analyzed the relationship between initial phosphate levels and the risk of mortality using receiver operating characteristic (ROC) curves. We and others have previously shown that phosphate can act as a prognostic marker in CA patients achieving ROSC [21,22]. Based on the initial analysis, we stratified OHCA patients into low- and high-risk groups with the respective cutoffs from the overall cohort, according to the Youden Index (YI). The primary endpoint of the study was the risk of mortality in high- and low-risk patients (stratified by phosphate) with and without MCS. We compared these analyses with the mortality of risk-stratified groups based on the calculated YI of lactate within the overall cohort as a known and established prognostic marker [18,19,28] routinely used in intensive care.

### 2.2. CPR and Mechanical Circulatory Support

Cardiopulmonary resuscitation was performed according to the 2015 guidelines of the European Resuscitation Council [29]. MCS was used if indicated by the individual decision of the attending physician. Two different types of MCS were used: Impella CP (Abiomed, Danvers, MA, USA) and Sorin Lifebox (Sorin Group, Munich, Germany). Subsequently, the patients were transferred to an intensive care unit, where target temperature management with a target of 34 °C was established for 24 h, followed by 72 h of normal body temperature.

### 2.3. Statistical Analysis

Demographic data were presented as means ± standard error of the mean (SEM). Differences between the groups were investigated by unpaired *t*-tests (normally distributed data, as indicated by the Kolmogorov–Smirnov test) or the Mann–Whitney test (non-normally distributed data). ROC analyses with the initial phosphate and lactate levels were performed to identify their prognostic value for in-hospital mortality. From the ROC curve, the YI of phosphate and lactate could be derived, which facilitated the classification of low- and high-risk patients. Survival was assessed by Kaplan–Meier analyses using the log-rank (Mantel–Cox) test to assess 30-day mortality. Significance was assumed if *p* < 0.05. The statistical analyses were performed in GraphPad Prism 9.3.0 for macOS/Windows.

## 3. Results

### 3.1. Study Population

**Figure 1:**
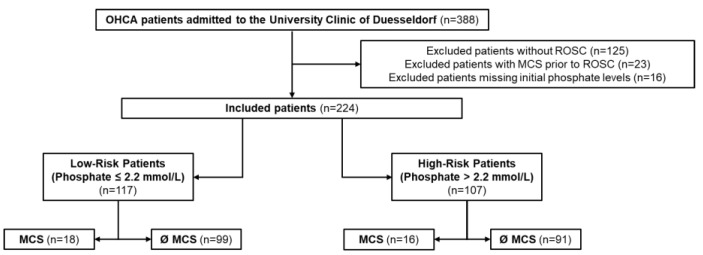
Flowchart representing screened and included OHCA patients with exclusion reasons. Stratification was performed according to the Youden Index.

Of the initially screened 388 OHCA patients, 125 did not reach ROSC, 23 received MCS before ROSC, and 16 had no recorded serum phosphate on admission. These patients were excluded a priori. The remaining 224 OHCA patients were included in the retrospective analysis (Figure 1).

**Figure 2:**
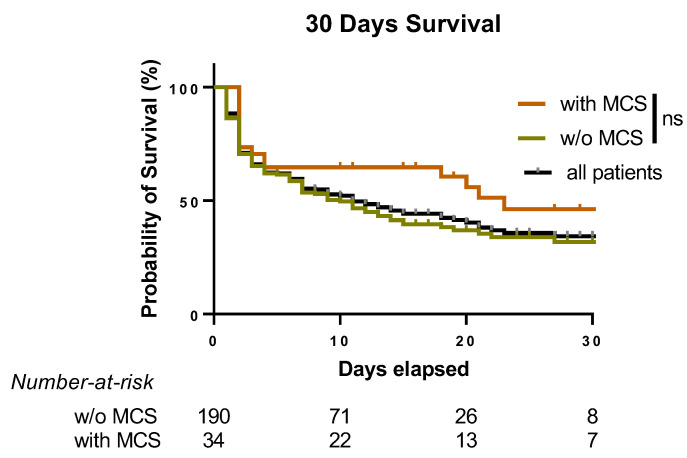
Comparison of survival in OHCA patients achieving ROSC with the following MCS. Kaplan–Meier curve representing the 30-day survival of OHCA patients ± MCS. All patients: *n* = 224, of which *w/o* MCS: *n* = 190 and with MCS: *n* = 34. Log-rank (Mantel-Cox) test: OHCA patients ± MCS *p* = 0.0611.

Overall and within the entire collective, MCS was not associated with a greater chance of survival (Figure 2).

The mean age of the OHCA patients was 65.6 (±0.98) years, with a higher proportion of males (65.9%, Table 1). In 39.8%, the interventionalist deemed acute myocardial infarction (AMI) the cause of CA. Bystander CPR was performed in 44%, and the mean time to ROSC was 27 ± 1.71 min. In total, 34 patients received MCS (15%). Patients surviving hospital discharge showed a lower time to ROSC (*p* < 0.0001) and higher proportions of an assumed AMI (52.6 vs. 27.1%, *p* = 0.0001), a witnessed CA (65.4 vs. 82.5%, *p* = 0.0061), and an initial shockable rhythm (73.6 vs. 38.9%, *p* < 0.0001) than deceased patients. The complete baseline characteristics of the entire collective are shown in Table 1.

### 3.2. Initial Phosphate and Lactate Level Elevations in Deceased OHCA Patients

**Figure 3:**
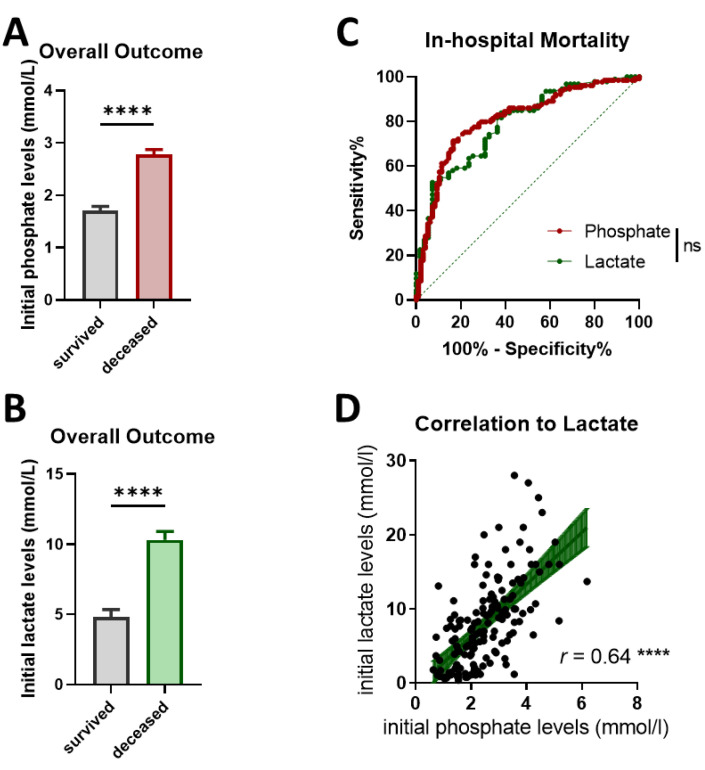
Initial serum phosphate and lactate level elevations identify OHCA patients with a higher risk of mortality following ROSC. Average phosphate (**A**) and lactate (**B**) levels in patients depending on the overall outcome during the hospital stay. (**C**) Comparison of ROC curves of serum lactate and phosphate levels to predict mortality. (**D**) Correlation between the initial serum lactate and phosphate levels (95% CI: 0.54–0.73, R^2^ = 0.41); ns *p* > 0.05, **** *p* < 0.0001.

Patients who died had a 1.6-fold higher average phosphate level than survivors (Figure 3A, *p* < 0.0001). In contrast, the average initial phosphate levels did not differ between patients with MCS (2.46 ± 0.16 mmol/L (SEM)) and those without MCS (2.3 ± 0.08 mmol/L, *p* = 0.2559). Similarly, the lactate levels of OHCA patients who died during the hospital stay were twice as high as the ones of surviving patients (Figure 3B, *p* < 0.0001).

ROC analyses on in-hospital mortality revealed good discriminations with comparable AUCs for both biomarkers (phosphate: AUC of 0.80, 95% CI: 0.73–0.86, *p* < 0.0001; lactate: AUC of 0.79, 95% CI: 0.71–0.85, *p* < 0.0001). However, among themselves, none of the parameters proved superior in mortality prediction (*p* = 0.74). Moreover, we could determine a moderate relationship between the two parameters by correlation analysis (Pearson *r* = 0.64, Figure 3D, *p* < 0.0001). The YI for phosphate and lactate were 2.2 mmol/L and 5.3 mmol/L, respectively, which we set as a cutoff for the risk stratification of the group comparison.

### 3.3. MCS Implementation in Low and High-Risk Patients Divided by Phosphate and Lactate

**Figure 4:**
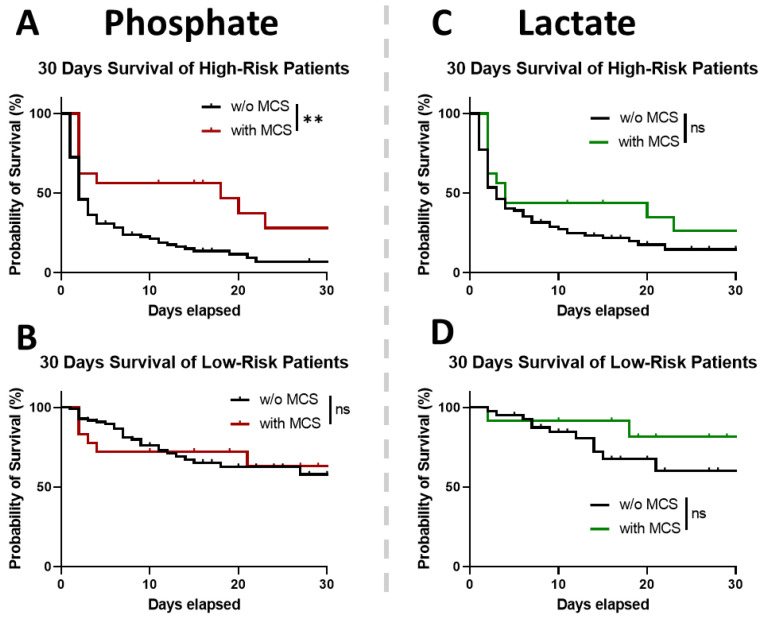
Overall outcome of OHCA patients ± MCS, stratified by Youden indices of initial phosphate and lactate levels. Kaplan–Meier curves representing 30 days survival of high-risk (**A**) or low-risk patients (**B**) assessed by the Youden Index of the initial phosphate levels (>2.2 mmol/L); high-risk patients: *w/o* MCS: *n* = 91; with MCS: *n* = 16. low-risk patients: *w/o* MCS: *n* = 99; with MCS: *n* = 18. Kaplan–Meier curves representing 30 days survival of high-risk (**C**) or low-risk patients (**D**) assessed by the Youden index of the initial lactate levels (>5.3 mmol/L); high-risk patients: *w/o* MCS: *n* = 83; with MCS: *n* = 16. low-risk patients: *w/o* MCS: *n* = 41; with MCS: *n* = 12. ns *p* > 0.05, ** *p* < 0.01.

In high-risk patients stratified by phosphate > 2.2 mmol/L, MCS was associated with a higher chance of survival (Figure 4A, HR 2.3, 95% CI: 1.4–3.7; *p* = 0.0037). High-risk patients with MCS were, on average, younger, had a higher proportion of AMI as the cause of the CA, and presented initially more often a shockable heart rhythm. The other baseline characteristics of these high-risk OHCA patients after achieving ROSC are shown in Table 2. In low-risk OHCA patients with initial phosphate levels ≤ 2.2 mmol/L, MCS was not associated with a greater chance of 30-day survival (assessed by a Kaplan–Meier test, *p* = 0.9385).

Lactate’s cutoff for risk stratification was 5.3 mmol/L. MCS did not affect the chance of survival in either low-risk (Figure 4C, *p* = 0.41) or high-risk OHCA patients (Figure 4D, *p* = 0.1345) stratified by the initial lactate levels.

## 4. Discussion

### Main Findings

This study shows evidence that initial phosphate and lactate level elevations following ROSC are similar in differentiating patients, in whom MCS was associated with a greater chance of survival. Even though both index biomarkers, lactate and phosphate, presented good discrimination to predict in-hospital mortality, our data suggested that phosphate was more suitable for risk stratification. To the best of our knowledge, this is the first study to investigate survival in OHCA patients with ROSC, who might benefit from MCS depending on their initial phosphate and lactate levels.

In line with several previous studies [18,19,21,22,28,30], we found that elevation of initial serum phosphate and lactate levels predicts higher mortality in OHCA patients who achieve ROSC. In our single-center collective, the initial phosphate levels showed good discrimination of higher mortality by ROC (Figure 3C, *p* < 0.0001), similar to the one in previously published studies on CA [21] and polytrauma patients [31]. The cutoff based on the YI (phosphate > 2.2 mmol/L) from the ROC analysis isolated high-risk OHCA patients, as proven by elevated 30-day mortality in this subgroup (Figure 4A, *p* = 0.0037). These patients seemed to benefit particularly from MCS implementation. In contrast, in low-risk patients, MCS showed no changes in the overall outcome (Figure 4B).

The initial lactate levels discriminated in-hospital mortality similarly well (Figure 3C, *p* < 0.0001). Several observational studies previously described an association between lactate levels and 30-day mortality in patients with cardiogenic shock treated with MCS [19,20]. In a recent Danish multicenter study, high lactate levels were identified as one of the reasons not to implement MCS in refractory OHCA patients [32], raising the question of whether their elevations would precisely distinguish between the patients who could profit from MCS. However, the study included only patients who did not achieve ROSC within the first 15 min of CPR [32], limiting the comparability to our collective. Surprisingly, we did not see a trend in the separation done according to the YI of lactate, as neither high-risk nor low-risk patients benefited from MCS (Figure 4C,D). A low sample size could perhaps explain this finding.

Following CA, several macro- and microcirculatory alterations and metabolic and coagulation disorders (defined altogether as a post-CA syndrome) [33] occur, mostly based on a cardio-circulatory imbalance [5]. These can even be worsened by post-CA hemodynamic instability after achieving ROSC, which is an often-encountered complication [5]. To countermand these changes and restore hemodynamic equilibrium, MCS devices have been increasingly used in patients with ongoing CA or predominant CS following AMI [10,34,35]. Evidence-based recommendations for MCS implementation are still imprecise. Especially the recently published ECMO-CS randomized controlled trial (RCT), which has shown no beneficial results. The routine use of MCS did not improve the mortality of patients with severe cardiogenic shock or deteriorating hemodynamics [16]. In line with this study, we also describe no generalizable benefit of using MCS in patients presenting with persistent shock after OHCA (Figure 2). However, several currently running RCTs, e.g., DanGer Shock (clinicaltrials.gov identifier: NCT01633502) or ECLS Shock (NCT03637205), aim to verify whether routine use of MCS in cardiogenic shock can aid the patient’s chance of survival as compared to conventional treatment. Currently, precise hemodynamic alterations in patients are measured invasively (e.g., by cardiac output monitoring by pulse contour analysis or a Swan-Ganz catheter) [13], which can sometimes have serious adverse effects [36]. Under these circumstances, including the initial serum phosphate levels (as feasible biomarkers) in this decision-making process might increase the precision of patients’ selection for MCS therapy. However, prospective studies must investigate phosphate’s independent prognostic value for MCS implementation.

The initial phosphate and lactate levels presented a moderate relationship (Figure 3D, Pearson *r* = 0.64, *p* < 0.0001). It is known that both parameters correlate with the time of ischemia [22,37,38], and they seem to have partially similar originating mechanisms for their plasmatic accumulation following CA [17,26] (which can explain this moderate relationship in our collective). Serum lactate increases due to anaerobic glycolysis as a source of energy production [17], whereas phosphate levels increase due to inadequate production of high-energy phosphates due to cell damage and reduced renal clearance. Since phosphate’s elimination occurs by the kidneys and ongoing ischemia and hypoxia result in the release of intracellular phosphate, a CPR-related kidney perfusion might induce a rise in serum phosphate levels, thereby reflecting reduced kidney perfusion. Future prospective studies should raise the question of whether the phosphate clearance would accurately reflect stable organ perfusion, as it may then be used as a marker for therapy monitoring during MCS.

Chronic kidney disease (CKD) may have influenced initial serum phosphate values and biased our results [39]. Creatinine did not differ between patients with and without MCS, yet it was higher in non-survivors than survivors. The difference vanished when calculating mean creatinine levels in patients with >2.2 mmol/L of initial serum phosphate (Table 2). Therefore, our main results should not be reasonably explained by the different stages of CKD.

In summary, within our collective, initial phosphate levels > 2.2 mmol/L stratified the OHCA patients who may have benefited from MCS. Phosphate elevations might more accurately reflect reduced organ perfusion in OHCA patients as one of the main casual reasons to implement MCS.

## 5. Limitations

Our study has several limitations. Due to its retrospective observational nature, no causal conclusions can be drawn. Second, the interventionalist indicated for or against MCS on an individual basis, which may have led to a selection bias. Third, some patients did not have documented lactate levels following hospital admission, which could perhaps explain why we saw an effect in the subgroup of phosphate but not lactate. On this basis, interpretations of the results on lactate levels should be analyzed carefully. Fourth, we did not consider the effect of acute/chronic kidney disease on phosphate elevations. Of note, other sources of phosphate have to be taken into account as well (e.g., phosphate buffer, bone marrow). As we cannot distinguish between these sources, their effects cannot be excluded from our analyses.

## 6. Conclusions

Our data show evidence for a significant association of survival with MCS therapy in patients with phosphate levels above 2.2 mmol/L (Youden Index) and for a similar discrimination of survival in OHCA patients following ROSC by lactate and phosphate. Given the current recommendations, clinicians may benefit from including serum phosphate levels in decision-making on MCS implementation. Prospective studies should assess the independent prognostic relevance of phosphate and its clearance (as it might accurately mirror organ perfusion) in the efficiency of MCS in patients with unstable hemodynamics.

## Figures and Tables

**Table 1:** Baseline characteristics of the studied population of OHCA patients achieving ROSC.

Mean ± SEM, *N* = 224	Total
*Study Population*	
age [years]	65.4 ± 0.99
gender [% male]	65.6
survival [%]	42.4
*Initial Laboratory Values*	
phosphate [mmol/L]	2.34 ± 0.07
creatinine [mg/dL]	1.5 ± 0.07
lactate [mmol/L] (*n* =154)	8.15 ± 0.47
potassium [mmol/L]	4.5 ± 0.07
LDH [U/L]	646.5 ± 57.8
NSE [µg/L] (*n* = 193)	103.6 ± 7.87
*CPR Data*	
MCS [%]	15.2
AMI [%]	37.1
bystander CPR [%]	44.8
initial rhythm (VT/VFib) [%]	49.6
time to ROSC [min]	25.2 ± 1.7
witnessed arrest [%]	71.9
compression-only CPR [%]	41

**Table 2:** Comparison of high-risk OHCA patients stratified by initial phosphate levels > 2.2 mmol/L (YI) with and without MCS after achieving ROSC.

Mean ± SEM, *n* = 107	MCS	*w/o* MCS	*p*-Value
*Study subpopulation*			
*n*	16	91	---
age [years]	55.5 ± 3.5	64.9 ± 1.65	0.0179
gender [% male]	68.75	71.43	0.7749
survival [%]	37.5	12.09	0.02
*Initial Laboratory Values*			
phosphate [mmol/L]	3.3 ± 0.17	3.2 ± 0.09	0.6510
creatinine [mg/dL]	1.3 ± 0.10	1.8 ± 0.14	0.3540
lactate [mmol/L] (*n* = 85)	9.8 ± 0.79	11.4 ± 0.72	0.3304
potassium [mmol/L]	4.2 ± 0.21	4.7 ± 0.13	0.1835
LDH [U/L]	582 ± 60	905 ± 134	0.5633
*CPR Data*			
AMI [%]	50	23.08	0.0347
bystander CPR [%]	43.75	46.15	>0.9999
initial rhythm (VT/VFib) [%]	81.25	35.16	0.0008
time to ROSC [min]	31.9 ± 5.6	36.1 ± 3.2	0.9983
witnessed arrest [%]	81.25	67.03	0.3806
compression-only CPR [%]	31.25	53.85	0.1110

## Data Availability

Data that support the findings of this study are available upon request from the corresponding authors.

## Abbreviations

AHA	American Heart Association
AMI	acute myocardial infarction
AUC	area under the curve
CKD	chronic kidney disease
CS	cardiogenic shock
(e)CPR	(extracorporeal) cardiopulmonary resuscitation
HR	hazard ratios
LDH	Lactate Dehydrogenase
LV-EF	left-ventricular ejection fraction
MCS	mechanical circulatory support
OHCA	out-of-hospital cardiac arrest
RCT	randomized controlled trial
ROC	receiver-operating curve
ROSC	return of spontaneous circulation
SCAI	Society for Cardiovascular Angiography and Interventions
SEM	standard error of the mean
YI	Youden Index
